# Effects of DNA Methylation on TFs in Human Embryonic Stem Cells

**DOI:** 10.3389/fgene.2021.639461

**Published:** 2021-02-23

**Authors:** Ximei Luo, Tianjiao Zhang, Yixiao Zhai, Fang Wang, Shumei Zhang, Guohua Wang

**Affiliations:** ^1^School of Computer Science and Technology, Harbin Institute of Technology, Harbin, China; ^2^Information and Computer Engineering College, Northeast Forestry University, Harbin, China

**Keywords:** DNA methylation, transcription factors, CTCF, CEBPB, H1-hESC

## Abstract

DNA methylation is an important epigenetic mechanism for gene regulation. The conventional view of DNA methylation is that DNA methylation could disrupt protein-DNA interactions and repress gene expression. Several recent studies reported that DNA methylation could alter transcription factors (TFs) binding sequence specificity *in vitro*. Here, we took advantage of the large sets of ChIP-seq data for TFs and whole-genome bisulfite sequencing data in many cell types to perform a systematic analysis of the protein-DNA methylation *in vivo*. We observed that many TFs could bind methylated DNA regions, especially in H1-hESC cells. By locating binding sites, we confirmed that some TFs could bind to methylated CpGs directly. The different proportion of CpGs at TF binding specificity motifs in different methylation statuses shows that some TFs are sensitive to methylation and some could bind to the methylated DNA with different motifs, such as CEBPB and CTCF. At the same time, TF binding could interactively alter local DNA methylation. The TF hypermethylation binding sites extensively overlap with enhancers. And we also found that some DNase I hypersensitive sites were specifically hypermethylated in H1-hESC cells. At last, compared with TFs’ binding regions in multiple cell types, we observed that CTCF binding to high methylated regions in H1-hESC were not conservative. These pieces of evidence indicate that TFs that bind to hypermethylation DNA in H1-hESC cells may associate with enhancers to regulate special biological functions.

## Introduction

DNA methylation is one type of epigenetic modification that plays an important role in many biological processes, including development, and disease progression (Das and Singal, 2004; Schübeler, 2015; Ambrosi et al., 2017; Horvath and Raj, 2018; Koch et al., 2018; Ando et al., 2019). Due to a recent technological development, mapping DNA methylation on a whole genome-wide scale has become less costly and more timesaving. While many genome-wide methylation patterns (methylomes) have been obtained in many physiological conditions, the mechanistic connections between DNA methylation changes and phenotypes are often missing.

The conventional view of the biological consequence of cytosine methylation is that it inhibits transcription factor (TF) occupancy to disrupt the protein-DNA interactions and thus represses the expression of the target genes (Lister et al., 2009; Neph et al., 2012; Thurman et al., 2012; Ambrosi et al., 2017). Many research groups have demonstrated that methylation on the binding sequence of a TF often abolished the *in vitro* interactions between the TF and its recognized DNA sequence (Hu et al., 2013; Zhu et al., 2016; Kribelbauer et al., 2017; Wang et al., 2018). However, in recent years, Kribelbauer et al. (2017) found that DNA methylation could increase p35 binding affinity *in vivo*. Hu et al. (2013) found that 47 proteins could bind to methylated CpG sites, with the majority showing a preference for specific DNA sequences. A recent large-scale *in vitro* survey on the interactions between proteins and methylated DNA sequences suggested that transcription factors (TFs) could change the sequence specificity with or without DNA methylation (Yin et al., 2017). In other words, a TF that recognizes an unmethylated DNA sequence could bind to another methylated DNA sequence. These new studies indicated that the previous studies of the effects of DNA methylation on protein-DNA interactions might have missed the correct methylated DNA sequences that could be recognized by TFs. Similarly, many previous studies suggested that TFs always interact with methylated DNA *in vivo* (Zhu et al., 2016; Wang et al., 2018). There are two possible explanations for the observation that TFs do not interact with methylated DNA sequences consistent with the conventional motif *in vivo* in the previous studies. One possibility is the intrinsic property of TFs which avoid methylated DNA sequences *in vivo*. The other possibility is that TFs are insensitive to the methylation status of the binding sequences. They do not interact with the methylated sequence in cells since there are no accessible methylated DNA sequences in most cells.

In this work, we performed a systematic analysis of the DNA methylation status at TF binding sites *in vivo*. This analysis took advantage of the availability of the large set of ChIP-seq data for TFs and WGBS for methylome in many cell types. By overlapping the *in vivo* TF binding sites and methylation levels in the same cell types, we obtained the methylation levels for each TF binding peak. According to whether the methylation level was greater than 0.6, we parted the peaks into two groups, one was hi-methyl and the other was low-methyl. Then two motifs were called for the two groups.

Using the two motifs, we located the binding site accurately. We further obtained the methylation level of the TF binding site. Interestingly, we observed that many TFs could bind to methylated CpG sites in different cells. We also observed that DNA methylation could alter the motifs slightly. We found that DNA methylation had a two-way effect, promoting some TF binding, and inhibiting other TF binding.

Interactively, TF binding could change the local DNA methylation. For example, the methylation near the CTCF binding sites showed an obvious reduction, indicating that CTCF may be involved in demethylation. In contrast, CEBPB and MAFK may maintain and even promote DNA methylation. Motivated by the distinct effects of TF binding on DNA methylation, we obtained the chromatin states of each TF binding peak. We found that TF hi-methyl binding always occurred at the enhancers, except for CTCF. Additionally, we systematically surveyed the DNA methylation-dependent CTCF and CEBPB binding in a variety of cell types. We found that the hi-methyl CTCF binding was unconservative. And we found that some DNase I hypersensitive sites, considered to be “open” and with high transcriptional activity regions, were also methylated in H1-hESC cells. This evidence indicated that TFs binding to hypermethylation DNA in H1-hESC cells may associate with enhancers to regulate special biological functions. We performed a deep analysis of two well-studied proteins, CTCF and CEBPB. It is widely considered that CTCF does not bind to methylated DNA, and the functions of CTCF are often DNA methylation dependent (Wang et al., 2012; Teif et al., 2014; Viner et al., 2016; Hashimoto et al., 2017). In our research, we found that CTCF could prevent methylation of CTCF target sites and was involved in passive demethylation. The methylated DNase I hypersensitive sites in H1-hESC, TF hi-methyl binding extensively at enhancers, and the unconservative hi-methyl bindings indicated that the protein-DNA methylation *in vivo* in H1-hESC cells may associate with enhancers to regulate special biological functions.

## Materials and Methods

### Data Access and Profiling

The WGBS datasets, ChIP-seq datasets, and DNase-seq datasets were download from the Encyclopedia of DNA Elements (ENCODE) project (Inoue et al., 2017; Kazachenka et al., 2018a,b). For the WGBS datasets, we retrieved two repetitions for each cell line. We downloaded the “bed” files which were produced with Bismark (Krueger and Andrews, 2011). Firstly, we merged two repetitions by summing up the count of reads in every loci. DNA methylation level was the ratio of the methylated reads covering the loci. For the ChIP-seq data, we downloaded “bed narrowPeak” files from ENCODE. The annotation of the datasets can be found in Supplementary Data 1. We filtered out the TFs with peak counts less than 500. For multiple experiments of one TF in the same cell, we took the one with the most peak counts for analysis. Our study included 1,200 TF ChIP-seq datasets and five WGBS datasets in five cell lines. The chromatin state segmentation annotations on four cell lines were download from the UCSC Genome Browser (Ernst and Kellis, 2017).

### WGBS and ChIP-Seq Data Integration

The analysis method is shown in Supplementary Figure 1. The average methylation level of the CpG sites aligning into a peak was calculated as the peak’s methylation level in each TF. Based on the distribution of peak methylation levels, it was found that many TFs could bind to methylated DNA regions. Then the peaks were classified into two groups based on whether the DNA methylation level was higher than 0.6. HOMER was used to call motifs from two groups, respectively (Heinz et al., 2010). It is worth noting that we only called the motifs of TFs which contained motifs and could bind hi-methyl DNA. Since DNA methylation may change the motif, the motifs obtained from the two sets of peaks may be different. We calculated the match score by using two motifs to scan the two group’s peaks, respectively. The position with the highest match score in the peak was the most likely binding site. We regarded it as the TF binding site. According to the methylation level on the relocated binding site, we could reconstruct a more accurate motif that directly binds to the methylation site.

## Results

### TFs Could Bind to Highly Methylated DNA in H1-hESC Cells

**FIGURE 1:**
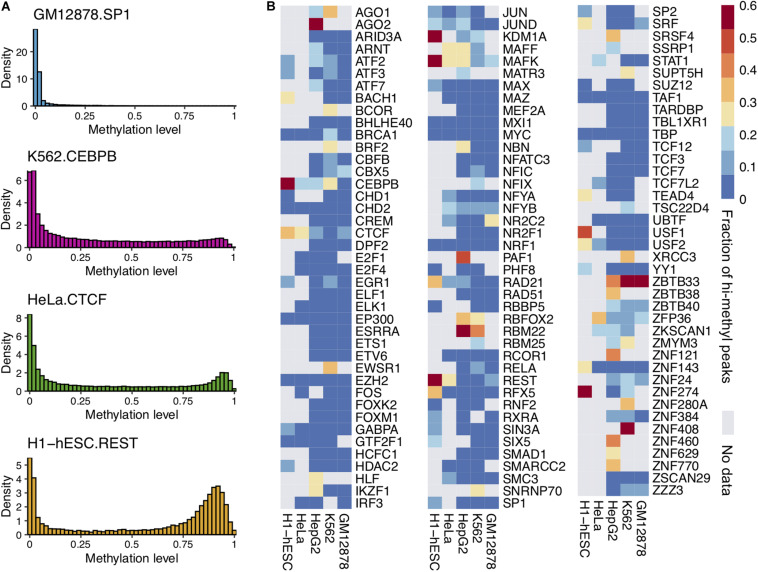
Some TFs could bind to highly methylated DNA regions and CpG sites. **(A)** The distribution of average DNA methylation levels for four TF peaks. **(B)** The heatmap of TF binding high methylated regions in five cell lines. The color represents the proportion of hi-methylated (methylation levels >0.6) peaks.

Previous studies suggested that some TFs could bind to methylated DNA *in vitro*. We wondered whether these TFs could bind to methylated DNA *in vivo*. For this purpose, we investigated the DNA methylation status of the DNA bound by the TFs. We superimposed the TF ChIP-seq and DNA methylome data from the same cell type. The average methylation level of all CpG sites within a ChIP-seq peak was calculated as the peak’s average methylation level. Then the distribution of the peaks’ average methylation levels for every TF was obtained. We performed the analysis of 1,200 ChIP-seq datasets on 786 TFs in five normal cell lines (GM12878, HepG2, HeLa, K562, and H1-hESC). Our results generalized the ability of 68 TFs to bind methylated DNA at the genome-scale though many TFs only bind to unmethylated sequences. For examples, as shown in Figure 1A, the far majority (98.89%) of SP1’s binding peaks’ average methylation level was less than 0.6 in GM12878.

It was sensitive to DNA methylation. On the other hand, some TFs (e.g., CEBPB, CTCF, and MAFK) could bind to methylated DNA. There were many peaks of these TFs with average methylation levels greater than 0.6. This observation was confirmed widely in multiple cell lines.

The peak with an average methylation level greater than 0.6 was considered as a hi-methylation binding region. The proportion of hi-methylation binding peaks to TF binding peaks was calculated. As Figure 1B shows, a substantial fraction of TF binding peaks were in hi-methylated regions. A total of 68 TFs showed clear tendency to bind to hi-methylated DNA regions among 786 TFs, and the fractions of these TF hi-methylation peaks were greater than 20%. The ratios of hi-methylated peaks to all 786 TFs are shown in Supplementary Figure 2. For example, CEBPB is known to bind methylated sequences based on *in vitro* binding assay. A total of 57.18% of CEBPB binding peaks were located in highly methylated regions in H1-hESC cells.

Interestingly, we found that DNA methylation patterns within TFBS can be cell specific. For instance, CEBPB, predominately binds to low methylated regions in the GM12878 cell line, while more than half of the CEBPB binding regions were hi-methylated in H1-hESC. In H1-hESC, we found that 15 TFs bind to hi-methylated DNA. These 15 genes may have great potential mediated by DNA methylation in the gene regulation of H1-hESC.

### DNA Methylation Has an Impact on TF Binding Motifs

**FIGURE 2:**
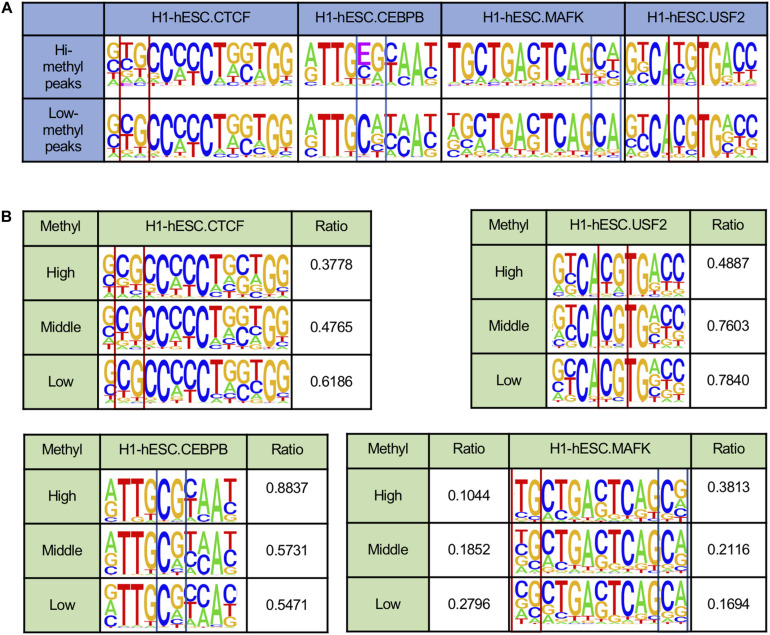
DNA methylation affects DNA binding sequences. *E indicates the methylated C. **(A)** TF binding motifs called from peaks with different DNA methylation states. **(B)** TF binding motifs in H1-hESC cell lines are rebuilt from binding sites with different DNA methylation states.

Several pieces of evidence suggest that DNA methylation could affect TF binding motifs (Zhu et al., 2016; Yin et al., 2017). Therefore, we scanned TF peaks to locate the binding sites and rebuild the motifs by using E to represent methylated C. As shown in Figure 2A, in H1-hESC cell lines, the two motifs of CTCF were similar. By comparing the proportion of peaks with CpGs on binding sites in the two peaks groups, there was still a significant difference. A total of 48.73% had low CTCF methylated peaks with CpGs, but only 25.34% had highly methylated peaks with CpGs. The opposite phenomenon appeared on the two motifs for CEBPB. We found that 59.20% had highly methylated peaks with CpG dinucleotides on the binding sites. However, only 35.13% had low methylation peaks with CpG dinucleotides on binding sites. MAFK had 18.41 and 15.47% peaks with CpGs in low and highly methylated regions, respectively. USF2 showed a significant increase in CpG ratio on binding sites between hi-methyl and low-methyl peaks, and the CpG ratios were 32.28 and 76.31%. The contradictory changes on CpG proportions give a hint about the two different mechanisms in DNA methylation affecting TF binding.

To further confirm that DNA methylation could affect the TF motifs in different ways, we calculated the methylation levels of each CpG site and the TF binding sites in H1-hESC cell lines (see Figure 2B). Here we only considered binding sites that contained CpG sites. Some binding sites may have contained multiple CpG sites. The maximum methylation levels at the CpG on the binding site were taken as the methylation level at the binding site. The binding sites were grouped based on the binding sites’ methylation level, >0.6 is high, <0.2 is low, and others are middle. Here we found the ratio of CpG at some sites increased as the methylation level decreased. For example, CTCF and USF2. There were also some sites where the proportion of CG decreased as the methylation level decreased. For instance, CEBPB and MAFK. Inconsistent with much research, DNA methylation had two different effects on the binding sites.

### TFs Bind to Hi-Methylation Related to the Whole Genome Methylation Level in H1-hESC

**FIGURE 3:**
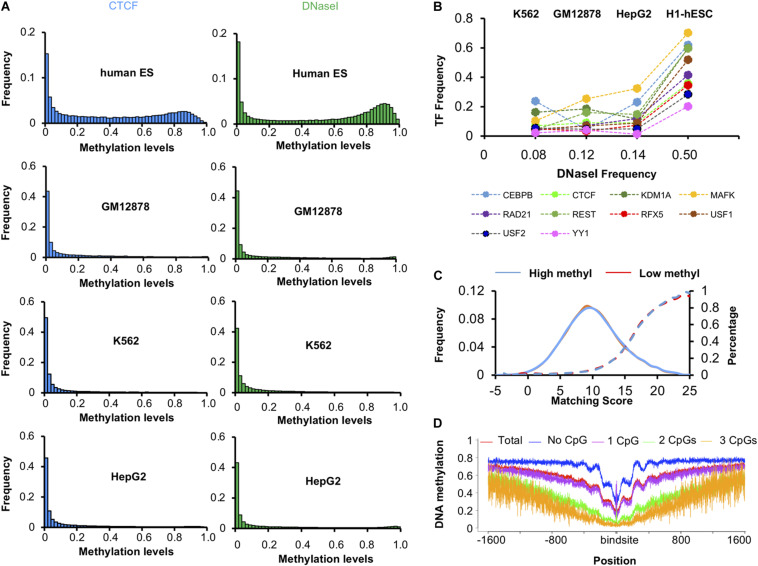
Methylation levels of TFs and DNase I region. **(A)** Distribution of methylation levels of CTCF and DNase I regions in four cell lines. **(B)** The ratio of highly methylated binding peaks and DNase I regions in four cell lines. **(C)** Matching score of CTCF in high methyl and low methyl peaks in H1-hESC cells. **(D)** DNA methylation level around CTCF binding sites.

We found that the DNA methylation levels of CTCF peaks in H1-hESC cell lines were significantly high. However, this phenomenon was absent in the other three cell lines. Then we calculated the distribution of DNA methylation levels in DHSs. As shown in Figure 3A, the methylation levels of CTCF peaks decreased synchronously with the methylation levels of DNase I regions in GM12878, HepG2, and K562. For further research, we calculated the ratio of methylated regions (>0.6) of DHSs and TF binding peaks in four cell lines. Unexpectedly, 50% of DNase I regions had been methylated in the H1-hESC cell line, while only 8, 12, and 14% had in K562, GM12787, and HepG2 cell lines, respectively. Then we checked the correlations of methylation levels of 10 TFs and DHSs in four cell types. As shown in Figure 3B, the ratio of methylated regions of TF peaks increased with the ratio of methylated regions (>0.6) of DHSs. When the DNase I methylation levels increased in H1-hESC cell lines, the TF’s methylation levels all increased synchronously. It was found that the methylation level of the TF binding site was associated with an increase in overall methylation levels.

We checked whether there was a difference between the binding sequence in high methyl and low methyl peaks. The match scores of known CTCF binding sequences to these peak regions were very similar (Figure 3C), suggesting that sequence difference was not the determinant of CTCF binding methylated DNA. Then the methylation level of 1600 bp upstream and downstream of the CTCF binding site were calculated (see Figure 3D). We found that the more CpGs on the binding sites, the lower methylation levels around the binding sites. This phenomenon is due to the fact that a higher level of CpG binding encourages more binding in the CpG island region. CpG-rich regions are thought to be probably never or only transiently methylated. We also found when CTCF binds to high methylation regions, there were periodic ripples in peripheral DNA methylation. Literature studies have shown that CTCF-PARP-1 interaction is related to demethylation. The presence of PARP-1 may protect CTCF-bound DNA sequences from being methylated by Dnmt1. These periodic ripples are associated with the interaction of PARP1 (Guastafierro et al., 2008; Kraus, 2008; Stadler et al., 2011; Thurman et al., 2012; Jubin et al., 2017). It suggests that CTCF can block methylation of a bound region and initiate passive demethylation binding in the highly methylated regions. As CTCF binding to methylated DNA was not due to the binding sequences, and the methylation levels of CTCF peaks increased with the overall methylation levels synchronously, the methylation may have hindered CTCF binding. We concluded that CTCF bound to methylated DNA *in vivo* for other reasons.

### TF Binding Promotes DNA Methylation and Triggers Demethylation

Some TFs could serve as readers of DNA methylation and changes to the DNA methylation states (Zhu et al., 2016; Yin et al., 2017). To explore the impact of TF binding on DNA methylation, we investigated the methylation level on both sides of the binding site. Here we used the two motifs to scan the hi-methyl and low-methyl peaks of the loci of the binding site. Then we calculated the methylation levels of 1600 bp upstream and downstream of the binding site.

**FIGURE 4:**
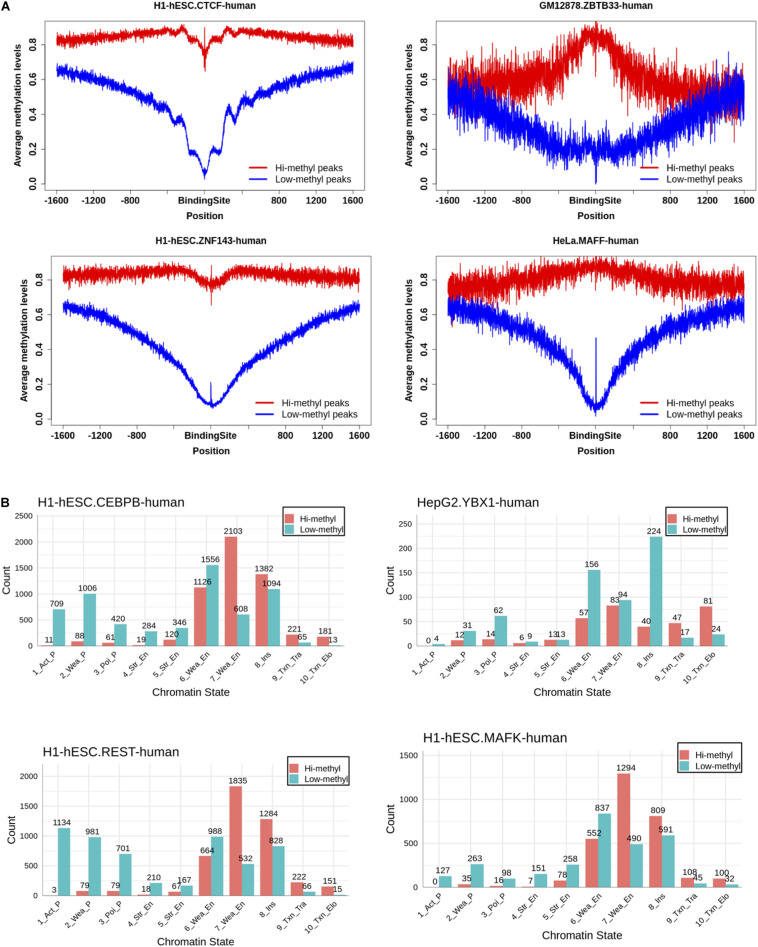
Investigation of the effect of TF binding on DNA methylation. **(A)** Distributions of DNA methylation level in the region of 1,600 bp away from the central of TF binding sites. TF binding could reduce the methylation level on the binding sites. While some could increase the methylation level on the binding sites. **(B)** The chromatin states of TF hi-methylation bindings are most at enhancers.

We were pleasantly surprised to find that the binding of TF had a two-way effect on the DNA methylation level. As Figure 4A shows, the methylation levels of the CTCF and ZNF143 binding sites in hi-methyl peaks showed a significant drop compared to the flank of the binding sites. Consistent with many studies, this observation indicated that CTCF may be involved in demethylation (Zheng et al., 2017; Ren and Zhao, 2019; Wiehle et al., 2019). ZNF143 also contributed to demethylation. In contrast, the methylation of ZBTB33 and the methylation of the binding sites showedd a huge increase. This observation shows that ZBTB33 is involved in *de novo* methylation, suggesting a potential role for ZBTB33 in heterochromatin priming (Hudson and Buck-Koehntop, 2018). And we also found that TF combined with different methylation levels had different effects. The methylation of the MAFF and ZBTB33 binding sites increased in hi-methylation peaks but decreased in the low-methyl peaks. Other TF profiles can be found in Supplementary Data 2.

Different effects of TF binding on methylation motivated us to find the function of the hi-methyl binding. We overlapped the TF binding to the chromatin states and found that almost all TF hi-methyl binding occurred at enhancers, not at promoters, except for CTCF (Figure 4B, Supplementary Data 3). Promoter DNA methylation has been associated with the stable silencing of gene expression. In comparison, enhancer methylation’s role in transcription is less well characterized.

This analysis confirmed that dynamic DNA methylation is driven by the balance between DNA methyltransferases and TF binding. And that TF methylation-dependent binding regulating the enhancer has great research potential.

### Conservation of CTCF and CEBPB Binding High Methylated Regions in H1-hESC

**FIGURE 5:**
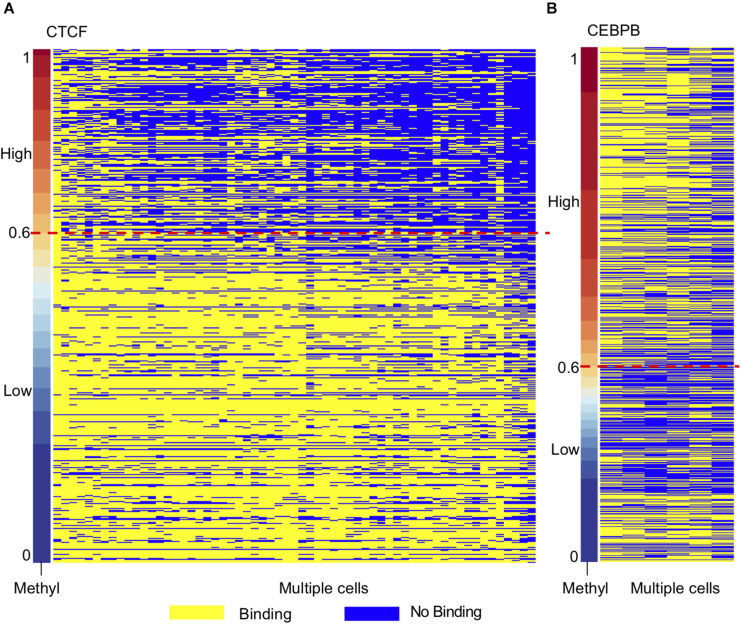
TF binding regions in H1-hESC show different conservatives in different methylation context. **(A)** Conservative of CTCF binding regions in H1-hESC. **(B)** Conservative of CEBPB binding regions in H1-hESC.

Motivated by the TF motifs effect on local DNA methylation profiles, we further investigated the conservation of TF binding across different cell types. As DNA methylation plays an essential role in embryonic development and methylation is relatively high at the whole genome level compared to other cells (Felsenfeld and Bell, 2000; Altun et al., 2010; Singer et al., 2014; Yizhar-Barnea et al., 2018), we used h1-hESC as the control group. Then we ordered the methylation levels of TF bound regions in H1-hESC cells and overlapped the regions in other cell lines. The conservatives of the bound regions across different cell types were found to be different between high methylated bound regions and low methylated bound regions. As Figure 5A shows, many CTCF high methylated bound regions were found in H1-hESC cells, while CTCF no longer binds in other cells. Low methylated bound regions had more conservatives than high methylated bound regions. We considered binding peaks in more than 80% of occupied cells to be conserved. A total of 18.73% of CTCF hi-methyl peaks were conserved. While in CTCF low-methyl peaks, 67.15% was conserved. It hinted that CTCF bound to DNA regions with different methylation have different biological functions. We extracted overlapped genes upstream and downstream of unconservative CTCF high methylated binding regions (methylation level >0.6) and low methylated binding regions (methylation level = 0.6) in 1000 bp separately. A total of 2,063 and 1,075 genes related to these two different methylated unconservative regions were obtained. GO enrichment analysis was performed on these genes, and we identified enriched biological processes related to these two gene groups. The five highest fold enrichment terms are shown in Table 1. The genes related to CTCF’s low methylated binding regions were enriched in calcium ion binding. While for CTCF’s high methylated binding regions, the genes were enriched to ATP binding, suggesting that in H1-hESC cells the methylated-dependent CTCF may be involved in regulating ATP binding.

**TABLE 1:** GO term enrichment for different methylation binding regions bound by CTCF and CEBPB.

	**Category**	**Term**	**Benjamini**	**Count**
CTCF binding in hi-methyl	MF	ATP binding	1.10E-05	220
	CC	Cytoskeleton	6.70E-06	73
	MF	Calcium ion binding	3.10E-05	120
	BP	Calcium ion transmembrane transport	1.40E-04	34
	MF	Rho guanyl-nucleotide exchange factor activity	4.00E-05	26
	MF	Actin binding	7.10E-05	58
	BP	Regulation of Rho protein signal transduction	3.00E-04	26
	MF	Calmodulin binding	9.60E-05	44
CTCF binding in low-methyl	BP	Homophilic cell adhesion via plasma membrane adhesion molecules	2.60E-10	37
	MF	Calcium ion binding	2.10E-07	80
	BP	Axon guidance	1.40E-04	28
	BP	Chemical synaptic transmission	3.80E-04	34
	MF	Extracellular-glutamate-gated ion channel activity	6.40E-04	9
	CC	Dendrite	3.30E-04	40
	MF	Ionotropic glutamate receptor activity	1.10E-03	8
CEBPB binding in hi-methyl	CC	Synapse	1.80E-04	26
	BP	Synapse assembly	7.30E-03	14
	BP	Ionotropic glutamate receptor signaling pathway	9.80E-03	9
	MF	Extracellular-glutamate-gated ion channel activity	4.60E-03	8
	CC	Plasma membrane	1.70E-03	245
CEBPB binding in low-methyl	BP	Nervous system development	1.50E-06	40
	BP	Cell adhesion	5.70E-04	46
	CC	Postsynaptic density	2.30E-04	25
	BP	Axon guidance	1.60E-03	23

As opposed to CTCF and CEBPB showed a different phenomenon (Figure 5B). We studied CEBPB binding across six cells. In contrast with CTCF, 41.91% of CEBPB hi-methyl peaks were conserved and 36.88% of CEBPB low-methyl peaks were conserved. The conservation levels in different methylation groups were similar. The hi-methyl bound regions in H1-hESC were always bound by CEBPB in other cell lines. We also carried out GO enrichment analysis as with CTCF. We observed that the gene related to methylated-dependent binding was regulating the extracellular-glutamate-gated ion channel activity in H1-hESC. Other TF profiles can be found in Supplementary Data 4.

## Discussion

Previous *in vitro* experiments found that TFs can bind to methylated sites. In this study, we analyzed the binding of TF to methylated DNA *in vivo* by integrating data from existing WGBS and ChIP-seq datasets. Many TFs were found that could bind to closed chromatin structure *in vivo*. Some of them could bind to methylated CpG directly. This phenomenon has not been discovered before because the phenomenon of TF binding to methylation mostly occurs in H1-hESC cell lines. In H1-hESC cells, the overall methylation level is higher than that of cell lines such as GM12878, HepG2, HeLa, and K562. We also found that some TFs bind to the methylated regions with the depletion of CpG at its binding site, such as CTCF. However, CEBPB is accompanied by the appearance of methylated CpG when it binds to methylated regions. So, DNA methylation affecting TF binding is bidirectional *in vivo*. Interactively, TF binding could change the local methylation bidirectionally. Such as ZBTB33 which involves *de novo* methylation. But CTCF and ZNF143, they could reduce the methylation levels on the flank of the binding sites. On regulation function analyses, TF hi-methylation binding sites were extensively located at enhancers. And the CTCF hi-methyl binding in H1-hESC was depleted in other cells. These pieces of evidence mean that DNA methylation may be involved in special gene regulation by the enhancer in H1-hESC.

Transcription factors with ChIP-seq data were studied in five cell lines. We found limitations when analyzing methylation at the TF binding site. In the follow-up analysis, we can only study the TFs with motifs. We ignored some TFs, such as Homez, without any binding motif. We only studied the TFs in which motifs could be identified, and focused on the analysis of CTCF and CEBPB in H1-hESC cells.

## Data Availability Statement

The original contributions presented in the study are included in the article/Supplementary Material, further inquiries can be directed to the corresponding author/s.

## Author Contributions

XL, TZ, YZ, FW, and SZ collected the data and did the analysis. XL, TZ, and FW drafted the manuscript. XL, YZ, and GW edited the manuscript. All authors read and approved the final manuscript.

## Conflict of Interest

The authors declare that the research was conducted in the absence of any commercial or financial relationships that could be construed as a potential conflict of interest.

## Supplementary Material

Supplementary Figure 1 The strategy of motif calling.

Supplementary Figure 2 The fraction of hi-methylated peaks of 1200 ChIP-seq profiles.

Supplementary Data 1 The annotation of TF and WGBS datasets included in this study.

Supplementary Data 2 Distributions of DNA methylation level in the region of 1600 bp away from the central of TF binding sites.

Supplementary Data 3 The chromatin states of TF hi-methylation binding.

Supplementary Data 4 TF binding regions in H1-hESC show different conservatives in different methylation contexts.
