# Supplementary figures and images for: Effects of DNA Methylation on TFs in Human Embryonic Stem Cells
Source: Front Genet. 2021 Feb 23;12:639461. doi: 10.3389/fgene.2021.639461 (PMC7940757; doi:10.3389/fgene.2021.639461)

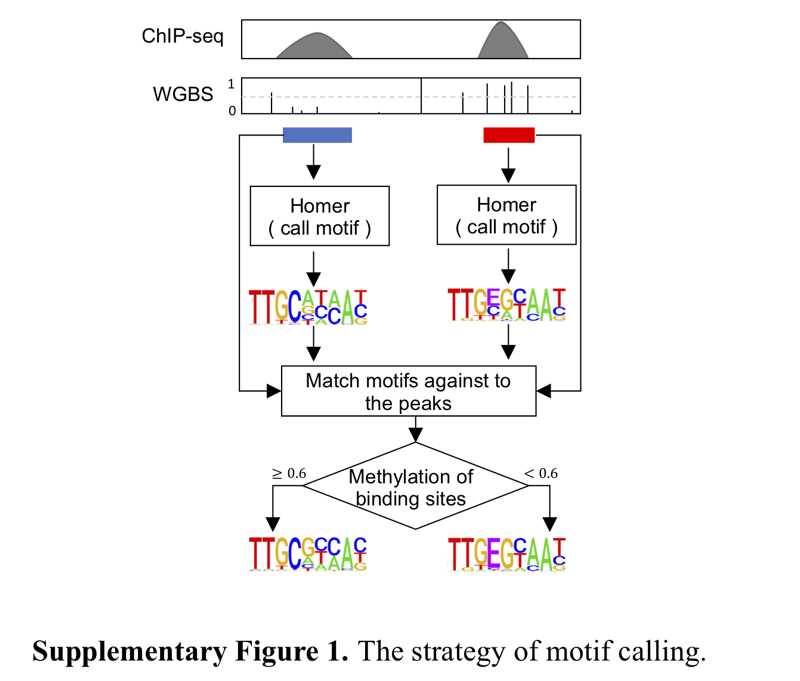

Supplement: Supplementary Figure 1 — The strategy of motif calling. [file Image_1.TIFF]

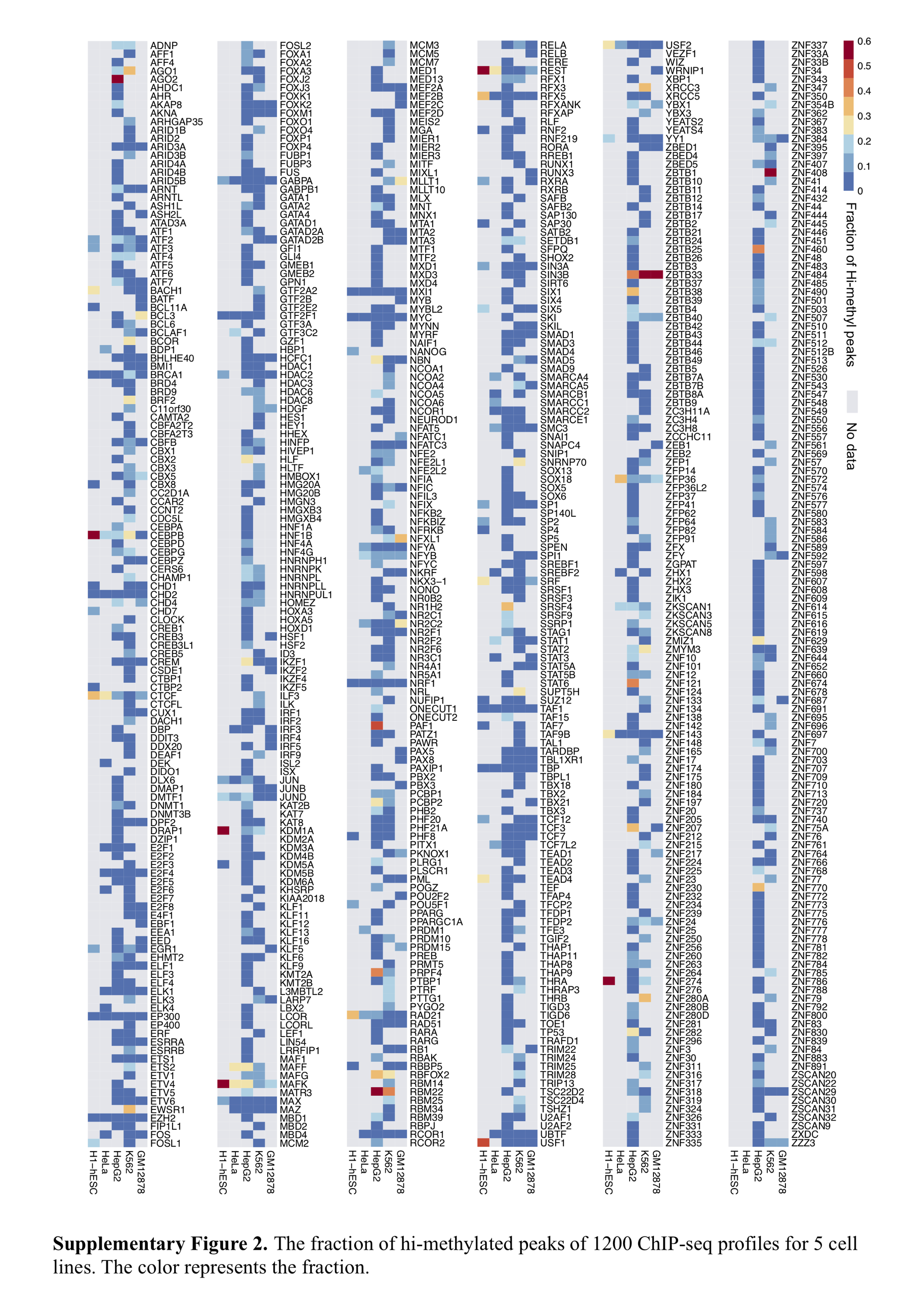

Supplement: Supplementary Figure 2 — The fraction of hi-methylated peaks of 1200 ChIP-seq profiles. [file Image_2.TIFF]

Supplementary Data 3. The chromation states of TFs hi-methylation bindings.

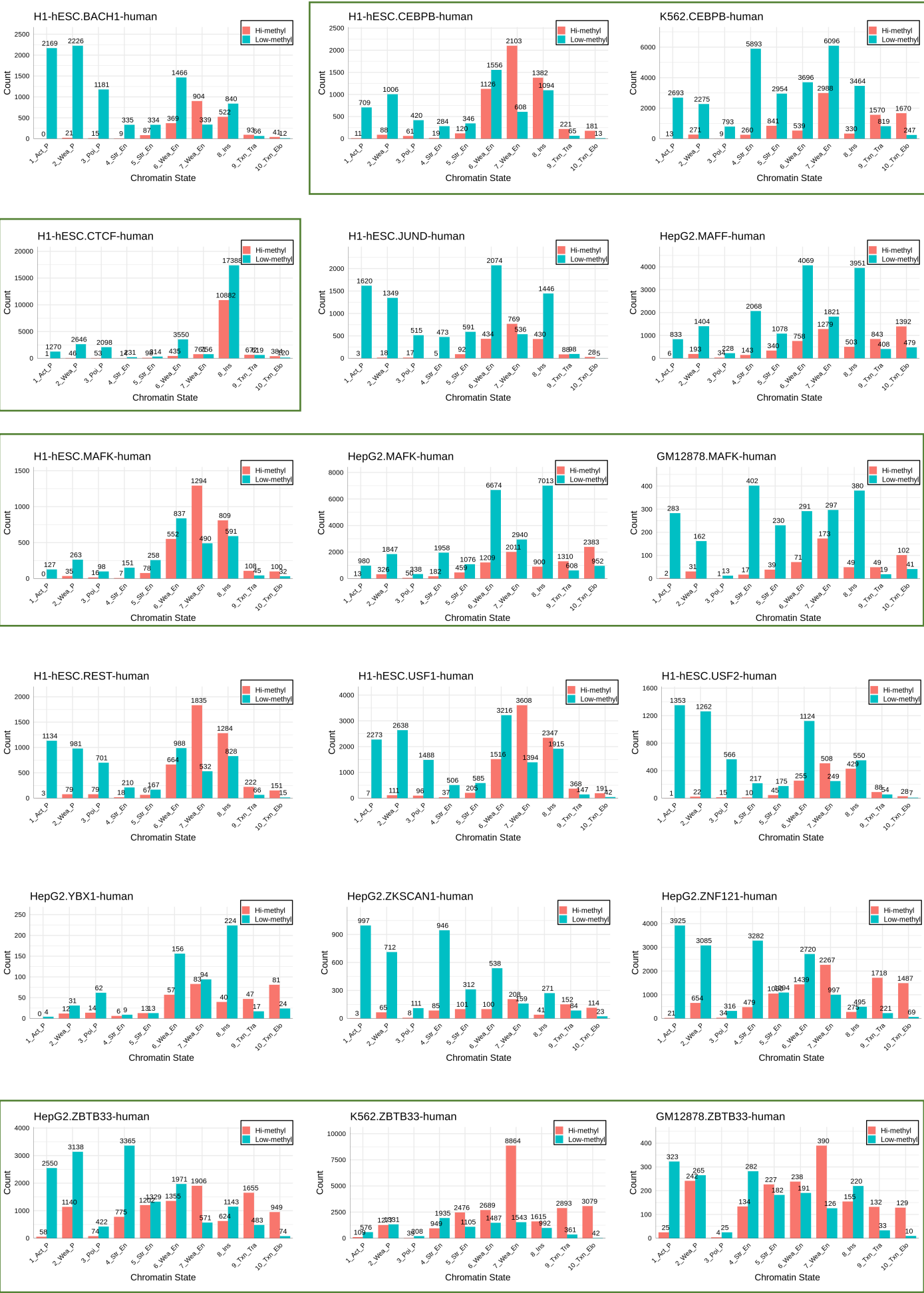

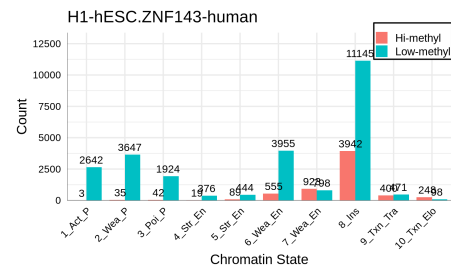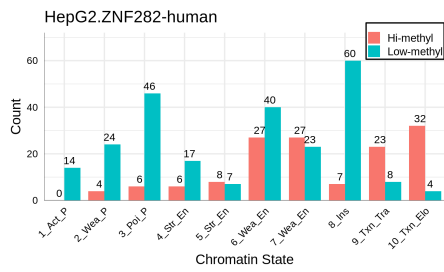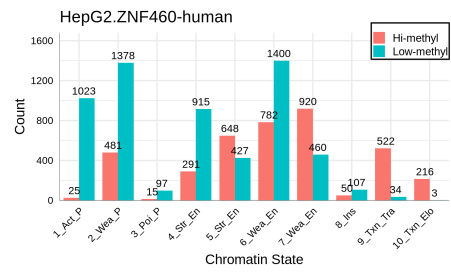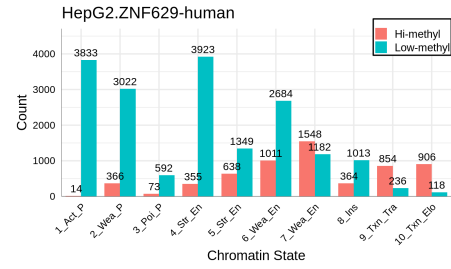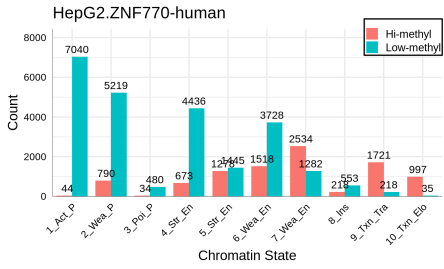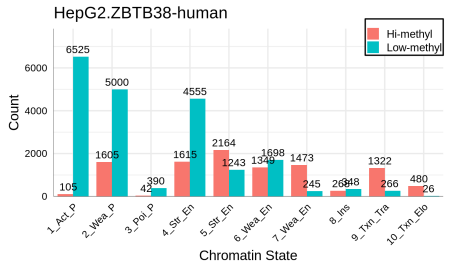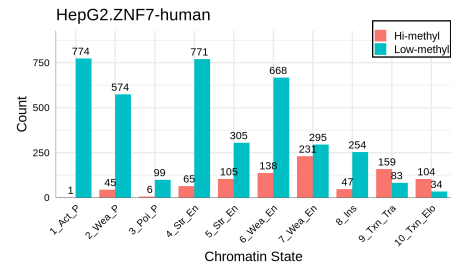

Supplement: Supplementary Data 3 — The chromatin states of TF hi-methylation binding. [file Data_Sheet_3.PDF]
