# Supplementary material for: Effects of DNA Methylation on TFs in Human Embryonic Stem Cells
Source: Front Genet. 2021 Feb 23;12:639461. doi: 10.3389/fgene.2021.639461 (PMC7940757; doi:10.3389/fgene.2021.639461)
Supplement: Supplementary Data 2 — Distributions of DNA methylation level in the region of 1600 bp away from the central of TF binding sites. [file Data_Sheet_2.PDF]

Supplementary Data 2. DNA methylation level in the region of 1600 bp away from the central of TFs' binding sites.

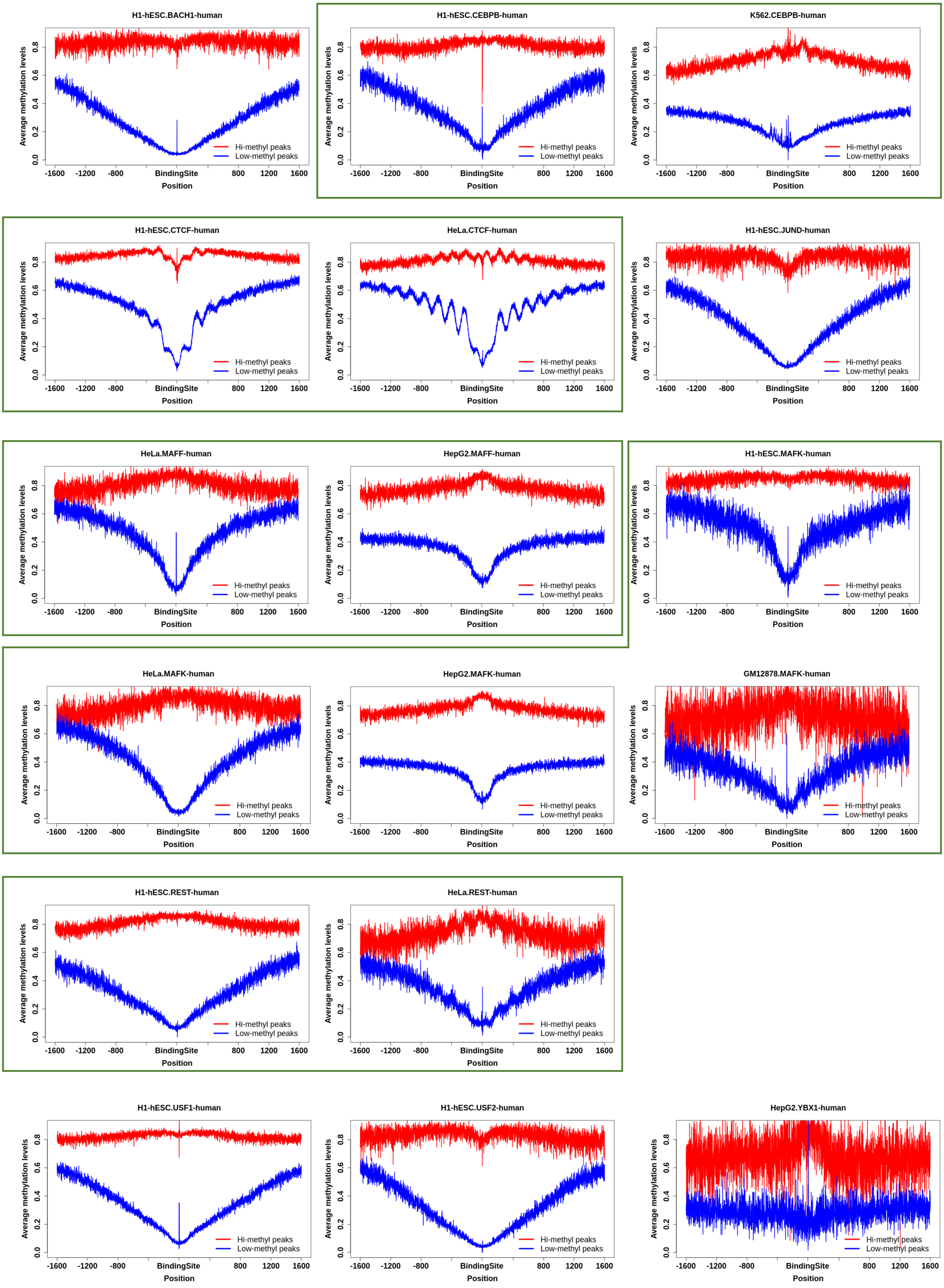

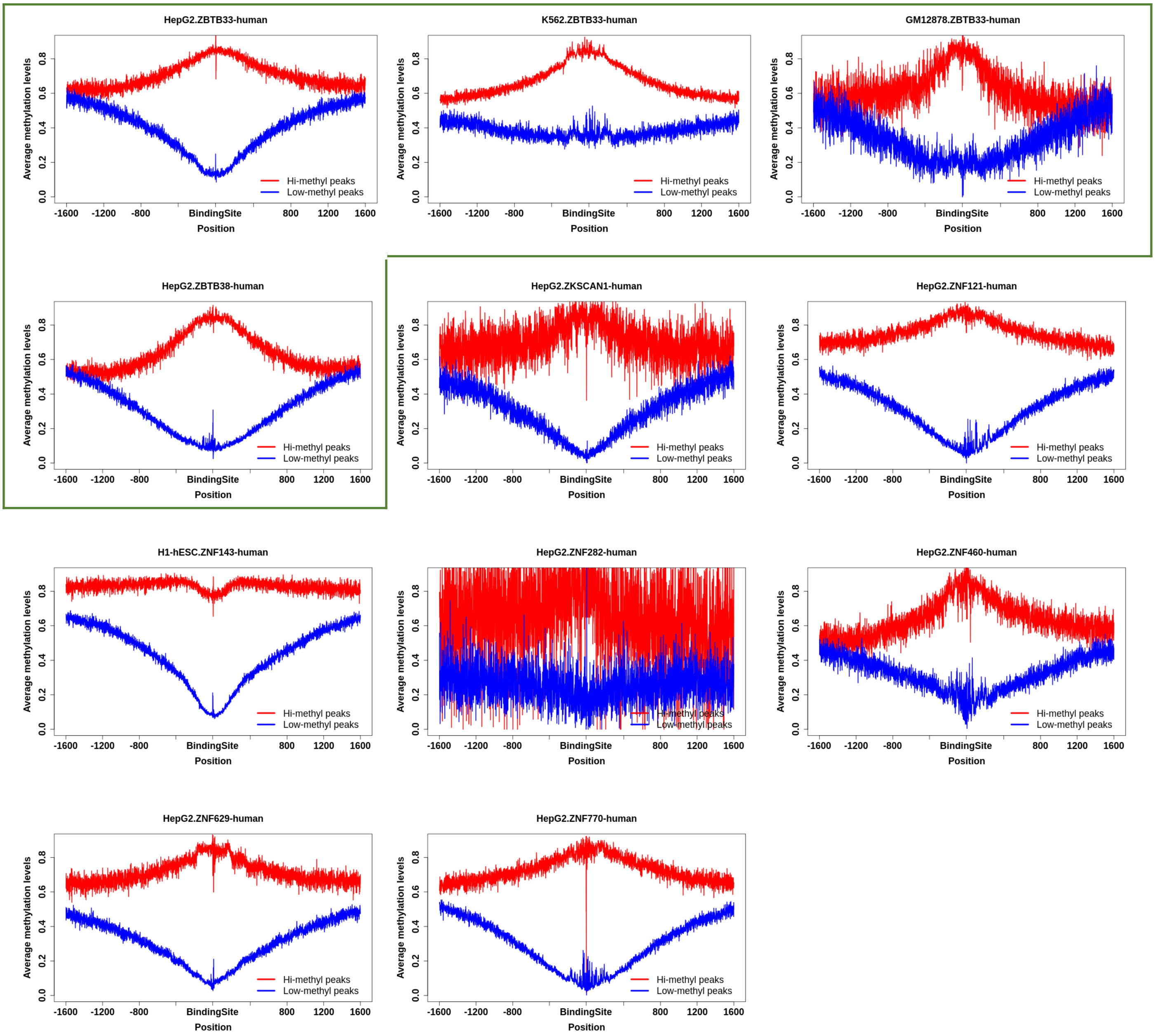
HepG2.ZBTB33-human
K562.ZBTB33-human
GM12878.ZBTB33-human
HepG2.ZBTB38-human
HepG2.ZKSCAN1-human
HepG2.ZNF121-human
H1-hESC.ZNF143-human
HepG2.ZNF282-human
HepG2.ZNF460-human
HepG2.ZNF629-human
HepG2.ZNF770-human
Average methylation levels
0.0
0.2
0.4
0.6
0.8
-1600
-1200
-800
BindingSite
800
1200
1600
Position
Hi-methyl peaks
Low-methyl peaks
