# Supplementary material for: Effects of DNA Methylation on TFs in Human Embryonic Stem Cells
Source: Front Genet. 2021 Feb 23;12:639461. doi: 10.3389/fgene.2021.639461 (PMC7940757; doi:10.3389/fgene.2021.639461)

Supplementary Data 4. TF's binding regions in H1-hESC show different conservative in different methylation context.

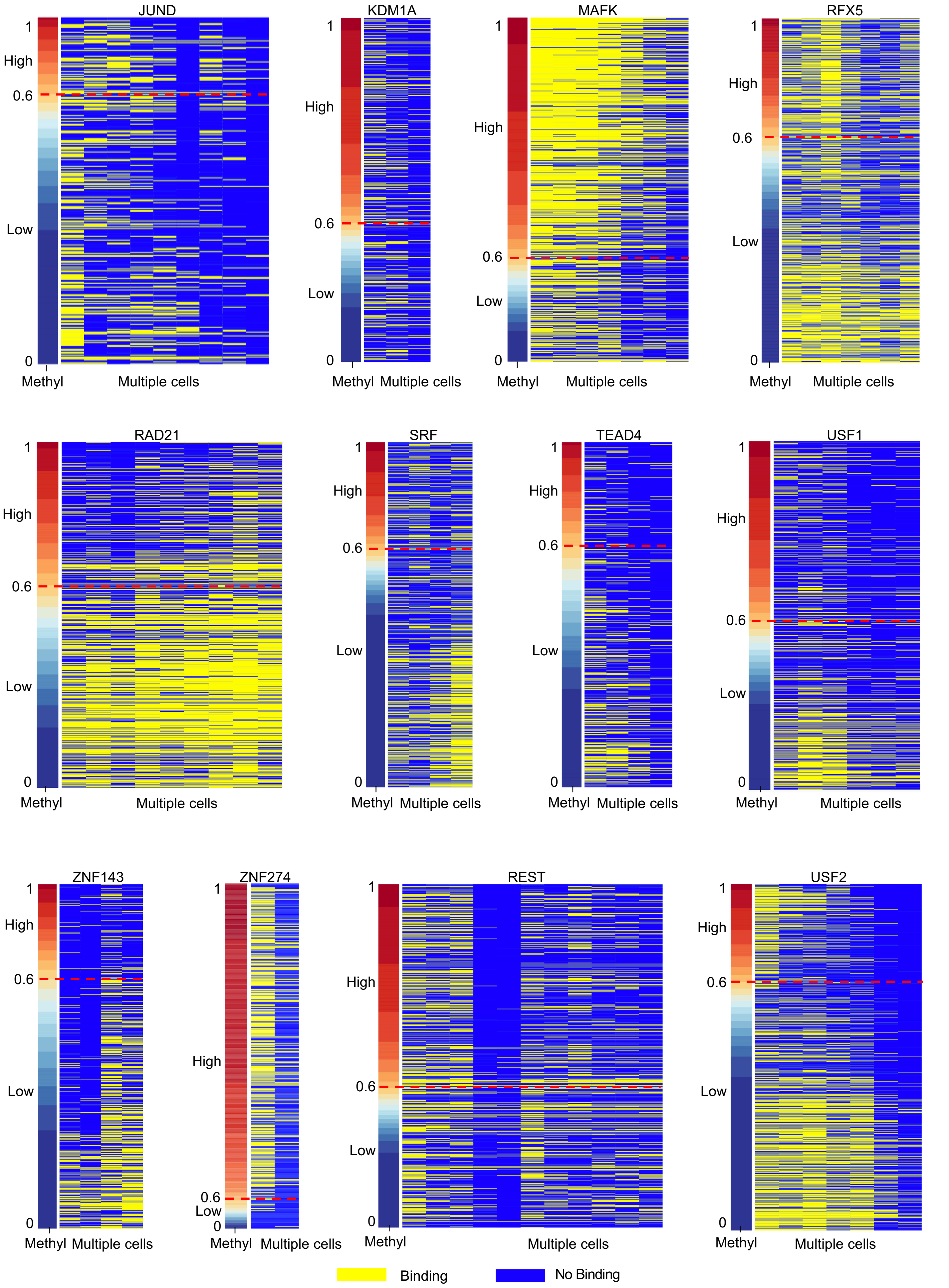

Supplement: Supplementary Data 4 — TF binding regions in H1-hESC show different conservatives in different methylation contexts. [file Data_Sheet_4.PDF]
